# Accuracy of the WSES classification system for pelvic ring disruptions: an international validation study

**DOI:** 10.1186/s13017-021-00399-4

**Published:** 2021-10-16

**Authors:** Szu-Han Wang, Chih-Yuan Fu, Francesco Bajani, Marissa Bokhari, Justin Mis, Stathis Poulakidas, Faran Bokhari

**Affiliations:** 1 Department of Trauma and Emergency Surgery, Chang Gung Memorial Hospital, Chang Gung University, Taipei, Taiwan; 2 Department of Trauma and Burn Surgery, Stroger Hospital of Cook County, Rush University, 8th Floor, 1950 West Polk Street, Chicago, IL 60612 USA

**Keywords:** Pelvic fracture, WSES guideline, Open pelvic fracture, Vascular injury

## Abstract

**Background:**

In 2017, a novel classification for pelvic injuries was established by the World Society of Emergency Surgery (WSES). We validated its effectiveness using nationwide real-world data. The roles of associated vascular injury and open fracture in this system were also evaluated.

**Methods:**

Patients with pelvic fractures in the National Trauma Data Bank 2015 dataset were retrospectively studied. First, the mortality rates were compared by WSES classification. Second, independent predictors of mortality were evaluated using a multivariate logistic regression model. Patients with and without associated vascular injuries and the same hemodynamic and pelvic ring stability statuses were compared. Patients with associated vascular injuries were compared to the proportion of nonsurvivors and survivors with unstable pelvic ring injuries. Third, the outcomes were compared between patients with open pelvic fracture and closed pelvic fracture in the mild, moderate and severe WSES classes.

**Results:**

During the 12-month study period, 44,163 blunt pelvic fracture patients were included. The mortality rates were 1.8%, 3.8% and 10.6% for the mild, moderate and severe WSES classes, respectively (*p* < 0.001). MLR analysis showed that unstable pelvic ring injury did not significantly affect mortality (*p* = 0.549), whereas open pelvic fracture and associated vascular injury were independent predictors of mortality (odds of mortality: open pelvic fracture 1.630, *p* < 0.001; associated vascular injury 1.602, *p* < 0.001). Patients with associated vascular injuries showed that there was no significant difference in the proportion of patients with unstable pelvic ring injuries between survivors and nonsurvivors (37.2% vs. 32.7%, *p* = 0.323). In all three classes, patients with open pelvic fractures had significantly higher mortality rates and infection rates than patients with closed fractures (mortality rates: minor 3.5% vs. 1.8%, *p* = 0.009, moderate 11.2% vs. 3.3%, *p* < 0.001, severe 23.8% vs. 9.8%, *p* < 0.001; infection rates: minor 3.3% vs. 0.7%, *p* < 0.001, moderate 6.7% vs. 2.1%, *p* < 0.001, severe 7.9% vs. 2.8%, *p* < 0.001).

**Conclusions:**

Based on this nationwide study, the WSES guideline provides an accurate and reproducible classification of pelvic fractures. It is recommended that open/closed fractures and associated vascular injuries be evaluated as supplements of the WSES classification.

## Background

Pelvic fracture is often a result of high-energy blunt trauma and accounts for approximately 3% of all skeletal injuries [1–3]. In addition to bony injury, which may result in pain and disability, the associated vascular injury could be life-threatening, with an overall mortality rate of 10–26% [4, 5]. Previously, pelvic fracture was classified using two classification systems, the Young and Burgess classification and the Tile classification, which are based only on anatomical injuries [6, 7]. In 2017, a novel classification and guideline were established by the World Society of Emergency Surgery (WSES), which includes surgeons from around the world [8]. This classification considered both physiological status and mechanical stability to enable more effective critical decision making.

In the current study, the effectiveness of the WSES classification for pelvic injuries was validated using nationwide real-world data. Although pelvic ring stability was evaluated by the WSES classification, associated vascular injury was not. It has been reported that the severity of vascular injury is a more significant factor in determining patient outcomes than the fracture pattern in patients with pelvic fracture [9]. Therefore, the role of vascular injury in this system was also analyzed. Finally, a subcohort stratification for patients with open pelvic fracture was performed. We tried to examine whether the outcomes of open and closed pelvic fracture patients with the same WSES injury severity are different.

## Methods

### A priori hypothesis

In the current study, we hypothesized that the WSES classification is accurate and reliable in the prediction of outcomes of patients with pelvic injuries.

### Dataset and time-window

Research Datasets of the National Trauma Data Bank (NTDB) 2015 were retrospectively queried.

### Inclusion/exclusion criteria

The inclusion criteria of the current study were patients with blunt pelvic fractures [International Classification of Diseases (ICD)-9-CM codes: 808.xx]. The exclusion criteria were patients with (1) penetrating trauma, (2) burns, (3) other or unknown trauma mechanisms or (4) severe head injuries [abbreviated injury scale (AIS) of the head score ≥ 3] that could affect trauma outcomes [10].

### Study setting

The data recorded in the NTDB, including age, sex, systolic blood pressure (SBP) in the emergency department (ED), pulse in the ED, respiratory rate (RR) in the ED, oxygen saturation in the ED, Glasgow Coma Scale (GCS) in the ED and injury severity score (ISS), were collected and evaluated. The covariates analyzed in the current study included (1) open (ICD-9-CM: 808.1, 808.3, 808.5, 808.5x, 808.9) and closed pelvic fracture, (2) unstable (ICD-9-CM: 808.43, 808.53) and stable pelvic ring injuries and (3) the presence and absence of associated vascular injuries (ICD-9-CM: 902.xx).

Unstable hemodynamics was defined as SBP < 90 mmHg, pulse > 120 or use of vasopressor. Pelvic fracture patients were classified based on the WSES guidelines (minor pelvic injuries: patients with stable hemodynamics and stable pelvic ring injuries; moderate pelvic injuries: patients with stable hemodynamics and unstable pelvic ring injuries; severe pelvic injuries: any patients with unstable hemodynamics) [8].

### Outcome measurements

Mortality was the primary outcome of the current study. Secondary outcomes included complications defined in the glossary of the NTDB [infection was present if any of the following were reported: severe sepsis (key = 32), superficial surgical site infection (key = 23), deep surgical site infection (key = 12), organ/space surgical site (key = 19) or osteomyelitis (key = 29); acute kidney injury (AKI), which is often related to sepsis or shock (key = 4)], hospital length of stay (LOS) and intensive care unit (ICU) LOS [11].

### Study design

Mortality rates were compared by WSES classification to validate the effectiveness of the classification system. The odds for mortality were then calculated using logistic regression analysis for all pelvic injury patients and patients with isolated pelvic injuries.

The characteristics of nonsurvivors and survivors were compared in all patients. Statistically significant variables in the univariate analysis were included in a multivariate logistic regression (MLR) model using the “enter method” for evaluation of independent predictors of mortality. Patients with and without associated vascular injuries and the same hemodynamic and pelvic ring stability statuses were also compared according to WSES classification. In patients with associated vascular injuries, the proportions of unstable pelvic ring injuries were compared between nonsurvivors and survivors. Finally, the outcomes (mortality, infection, AKI, hospital LOS and ICU LOS) were compared between patients with open pelvic fracture and closed pelvic fracture by mild, moderate and severe WSES class. Patients were then divided into open and closed pelvic fracture groups, and the mortality rates were compared by WSES classifications with linear trend analysis.

### Statistical analysis

All original files from the NTDB were merged and analyzed with R software, version 3.5.0 from R Core Team (R Foundation for Statistical Computing, Vienna, Austria, 2018) and R Studio software, version 1.1.453 from R Studio: Integrated Development for R (R Studio, Inc., Boston, Massachusetts, 2016) [12]. Nominal data are presented as numbers and percentages and were compared using chi-square tests, and numerical data are presented as the means with standard deviations and were compared using Student’s *t* tests. A value of *p* < 0.05 was considered statistically significant.

## Results

During the 12-month study period, 55,588 pelvic fracture patients from the NTDB were evaluated. A total of 11,425 patients had concomitant severe head injuries (AIS of the head score ≥ 3) or nonblunt trauma mechanisms (penetrating trauma, burn, other or unknown trauma mechanism) and were excluded. Therefore, 44,163 blunt pelvic fracture patients were included in the current study. The number of patients and proportion of WSES minor pelvic injuries, moderate pelvic injuries and severe pelvic injuries were 37,785 (75.6%), 3,638 (8.2%) and 2,740 (6.2%), respectively. Among all studied patients, 830 (1.9%) patients had open pelvic fractures, 4,184 (9.5%) patients had unstable pelvic ring injuries, and 975 (2.2%) patients had associated vascular injuries. The overall mortality rate was 2.5% (*N* = 1108).

Table 1 shows that the mortality rates and associated odds of mortality increased significantly as the class of injury increased from minor injury to severe injury in all pelvic injury patients (*N* = 44,163) and patients with isolated pelvic injuries (*N* = 30,863) (all patients: mortality rate: 1.8% in minor injuries, 3.8% in moderate injuries and 10.6% in severe injuries, *p* < 0.001; odds of mortality: 2.194 in moderate injuries and 6.513 in severe injuries with minor injuries as the baseline, *p* < 0.001) (patients with isolated pelvic injuries: mortality rate: 1.4% in minor injuries, 3.1% in moderate injuries and 8.1% in severe injuries, *p* < 0.001; odds of mortality: 2.215 in moderate injuries and 6.170 in severe injuries with minor injuries as the baseline, *p* < 0.001).

**Table 1:** Comparison of mortality rates and associated odds of mortality among different WSES classes of pelvic injuries in the NTDB 2015

Outcomes	Minor (*N* = 37,785)	Moderate (*N* = 3638)	Severe (*N* = 2740)	*p* value
All pelvic injury patients (*N* = 44,163)				
Mortality rate (*N*, %)	677 (1.8%)	140 (3.8%)	291 (10.6%)	< 0.001*
Odds of mortality	(Baseline)	2.194 (1.823–2.640)	6.513 (5.643–7.517)	< 0.001^†^

Outcomes	Minor (*N* = 25,046)	Moderate (*N* = 2074)	Severe (*N* = 3743)	*p* value
Patients with isolated pelvic injuries (*N* = 30,863)				
Mortality rate (*N*, %)	355 (1.4%)	64 (3.1%)	305 (8.1%)	< 0.001*
Odds of mortality	(Baseline)	2.215 (1.691–2.901)	6.170 (5.273–7.220)	< 0.001^†^

Mortality rate: *N* (percentage), odds of mortality: odds (95% confidence interval)

WSES = World Society of Emergency Surgery, NTDB = National Trauma Data Bank

*Chi-square test, ^†^Logistic regression

In the univariate analyses, a significantly higher proportion of open pelvic fracture (7.5% vs. 1.7%, *p* < 0.001), unstable pelvic ring injury (20.9% vs. 9.2%, *p* < 0.001) and associated vascular injury (10.9% vs. 2.0%, *p* < 0.001) was present in nonsurvivors (*N* = 1108) than in survivors (*N* = 43,055) (Table 2). However, the subsequent MLR analysis showed that unstable pelvic ring injury did not significantly affect mortality (*p* = 0.549) after adjusting for other covariates. Open pelvic fracture and associated vascular injury were independent predictors of mortality (odds of mortality: open pelvic fracture 1.630, *p* < 0.001; associated vascular injury 1.602, *p* < 0.001) (Table 3).

**Table 2:** Comparisons between nonsurvivors and survivors in all patients with pelvic fractures (*N* = 44,163)

Variables	Nonsurvivors (*N* = 1108)	Survivors (*N* = 43,055)	*p* value
Age	42.0 ± 57.4	40.4 ± 45.4	0.242*
Age ≥ 65 (*N*, %)	514 (46.4%)	13,687 (31.8%)	< 0.001^†^
Male (*N*, %)	673 (60.7%)	21,762 (50.5%)	< 0.001^†^
SBP (mmHg)	109.1 ± 46.4	131.1 ± 33.2	< 0.001*
Pulse (/min)	93.1 ± 36.0	87.6 ± 24.3	< 0.001*
Respiratory rate (/min)	18.2 ± 9.5	18.3 ± 5.9	0.748*
Oxygen saturation (%)	81.3 ± 33.7	90.6 ± 24.0	< 0.001*
GCS	10.8 ± 5.6	13.6 ± 4.2	< 0.001*
ISS	21.7 ± 15.8	10.8 ± 8.8	< 0.001*
Open pelvic fracture (*N*, %)	83 (7.5%)	747 (1.7%)	< 0.001^†^
Unstable pelvic ring injury (*N*, %)	232 (20.9%)	3,952 (9.2%)	< 0.001^†^
Associated vascular injury (*N*, %)	121 (10.9%)	854 (2.0%)	< 0.001^†^

Numerical data: mean (standard deviation); Nominal data: *N* (percentage)

SBP = systolic blood pressure, GCS = Glasgow Coma Scale, ISS = injury severity score

*Student *t* test, ^†^Chi-square test

**Table 3:** Multivariate logistic regression analysis for the evaluation of independent risk factors for mortality in all pelvic fracture patients (N = 44,163)

Variables	*p* value*	Odds of mortality	95% CI	
			Lower	Upper
Age ≥ 65	< 0.001	4.045	3.519	4.650
Male	< 0.001	1.374	1.205	1.567
SBP (mmHg)	< 0.001	0.991	0.989	0.993
Pulse (/min)	< 0.001	1.012	1.100	1.014
Oxygen saturation (%)	< 0.001	0.996	0.993	0.998
GCS	< 0.001	0.936	0.926	0.947
ISS	< 0.001	1.062	1.057	1.068
Open pelvic fracture	< 0.001	1.639	1.242	2.163
Unstable pelvic ring injury	0.549	–	–	–
Associated vascular injury	< 0.001	1.602	1.269	2.022

SBP = systolic blood pressure, GCS = Glasgow Coma Scale, ISS = injury severity score, CI = confidence interval

*Multivariate logistic regression

Furthermore, in patients with the same hemodynamic and pelvic ring stability statuses, patients with associated vascular injuries had significantly worse outcomes (higher mortality rates, longer hospital LOS and longer ICU LOS) than patients without associated vascular injuries (WSES minor injury: stable pelvic ring; WSES moderate injury: unstable pelvic ring) (Table 4). In contrast, a subgroup analysis of patients with associated vascular injuries showed that there was no significant difference in the proportion of unstable pelvic ring injuries between survivors and nonsurvivors (37.2% vs. 32.7%, *p* = 0.323) (Table 5).

**Table 4:** Comparisons between patients with and without associated vascular injuries in patients with the same hemodynamic and pelvic ring stability statuses (minor WSES class: stable hemodynamics and stable pelvic ring injury; moderate WSES class: stable hemodynamics and unstable pelvic ring injury)

Outcomes	Vascular injury (+)	Vascular injury (−)	*p* value
Minor (*N* = 37,785)	N = 508	N = 37,277	
Mortality (*N*, %)	50 (9.8%)	627 (1.7%)	< 0.001^†^
LOS (day)	13.7 ± 13.8	7.0 ± 7.8	< 0.001*
ICU LOS (day)	6.6 ± 9.3	0.7 ± 4.7	< 0.001*
Moderate (*N* = 3638)	N = 219	N = 3,419	
Mortality (*N*, %)	29 (13.2%)	111 (3.2%)	< 0.001^†^
LOS (day)	19.9 ± 23.3	11.1 ± 12.2	< 0.001*
ICU LOS (day)	9.7 ± 13.3	3.1 ± 7.2	< 0.001*

Numerical data: mean (standard deviation); Nominal data: N (percentage)

WSES = World Society of Emergency Surgery, LOS = length of stay, ICU = intensive care unit

*Student *t* test, ^†^Chi-square test

**Table 5:** Comparisons between nonsurvivors and survivors in pelvic fracture patients with associated vascular injuries (*N* = 752)

Variables	Nonsurvivors (*N* = 121)	Survivors (*N* = 854)	*p* value
Age	42.8 ± 44.4	42.2 ± 34.2	0.864*
Age ≥ 65 (*N*, %)	36 (29.8%)	193 (22.6%)	0.082^†^
Male (*N*, %)	93 (76.9%)	557 (65.2%)	0.011^†^
SBP (mmHg)	103.8 ± 42.1	109.2 ± 38.7	0.156*
Pulse (/min)	109.9 ± 36.5	96.9 ± 31.4	< 0.001*
Respiratory rate (/min)	19.8 ± 10.5	19.0 ± 8.1	0.332*
Oxygen saturation (%)	81.2 ± 34.3	88.5 ± 27.4	0.008*
GCS	9.9 ± 5.6	12.9 ± 4.3	< 0.001*
ISS	35.3 ± 13.5	26.2 ± 12.7	< 0.001*
Open pelvic fracture (*N*, %)	19 (15.7%)	73 (8.5%)	0.012^†^
Unstable pelvic ring injury (*N*, %)	45 (37.2%)	279 (32.7%)	0.323^†^

Numerical data: mean (standard deviation); Nominal data: *N* (percentage)

SBP = systolic blood pressure, GCS = Glasgow Coma Scale, ISS = injury severity score

*Student *t* test, ^†^Chi-square test

**Fig. 1:**
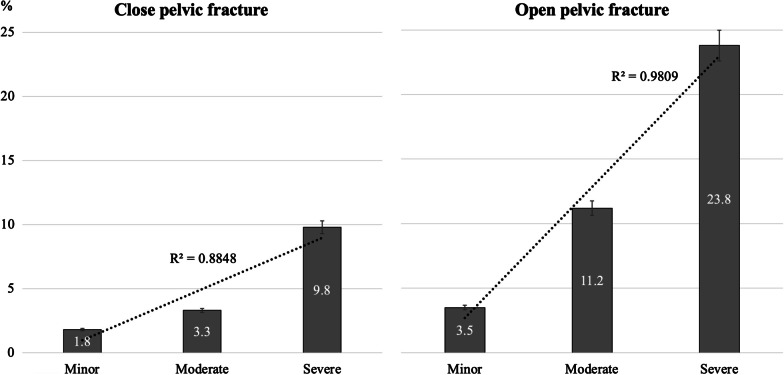
Mortality rates by WSES classes in open and closed pelvic fractures

Table 6 shows comparisons of open and closed pelvic fractures according to minor, moderate and severe classes of the WSES classification. In all three classes, patients with open pelvic fractures had significantly higher mortality rates and infection rates than patients with closed fractures (mortality rates: minor 3.5% vs. 1.8%, *p* = 0.009, moderate 11.2% vs. 3.3%, *p* < 0.001, severe 23.8% vs. 9.8%, *p* < 0.001; infection rates: minor 3.3% vs. 0.7%, *p* < 0.001, moderate 6.7% vs. 2.1%, *p* < 0.001, severe 7.9% vs. 2.8%, *p* < 0.001). In the moderate and severe classes, the proportion of patients with AKI in patients with open fractures was significantly higher than that in patients with closed fractures (moderate 1.5% vs. 0.4%, *p* = 0.022, severe 2.4% vs. 0.5%, *p* = 0.004). The hospital LOS and ICU LOS associated with open pelvic fractures were also significantly longer than those associated with closed pelvic fractures in all three classes. When patients were divided into open fracture and closed fracture groups, the mortality rates increased significantly with WSES classification in both groups. However, the R square from the linear trend analysis was larger in the open pelvic fracture group (*R*^2^ = 0.9809) than in the closed pelvic fracture group (*R*^2^ = 0.8848) (Fig. 1).

**Table 6:** Comparisons between open and closed pelvic fracture for the minor, moderate and severe WSES classifications (*N* = 44,163)

Outcomes	Open fracture	Closed fracture	*p* value
Minor (*N* = 37,785)	*N* = 398	*N* = 37,387	
Mortality (*N*, %)	14 (3.5%)	663 (1.8%)	0.009^†^
Infection (*N*, %)	13 (3.3%)	273 (0.7%)	< 0.001^†^
AKI (*N*, %)	2 (0.5%)	127 (0.3%)	0.580^†^
LOS (day)	11.8 ± 14.6	7.0 ± 7.9	< 0.001*
ICU LOS (day)	3.3 ± 7.3	0.7 ± 4.8	< 0.001*
Moderate (*N* = 3638)	*N* = 268	*N* = 3370	
Mortality (*N*, %)	30 (11.2%)	110 (3.3%)	< 0.001^†^
Infection (*N*, %)	18 (6.7%)	70 (2.1%)	< 0.001^†^
AKI (*N*, %)	4 (1.5%)	15 (0.4%)	0.022^†^
LOS (day)	16.6 ± 24.0	11.2 ± 12.0	< 0.001*
ICU LOS (day)	6.8 ± 11.6	3.2 ± 7.5	< 0.001*
Severe (*N* = 2740)	*N* = 164	*N* = 2,576	
Mortality (*N*, %)	39 (23.8%)	252 (9.8%)	< 0.001^†^
Infection (*N*, %)	13 (7.9%)	72 (2.8%)	< 0.001^†^
AKI (*N*, %)	4 (2.4%)	14 (0.5%)	0.004^†^
LOS (day)	15.5 ± 18.9	10.8 ± 13.4	< 0.001*
ICU LOS (day)	6.3 ± 9.7	4.1 ± 9.4	< 0.001*

Numerical data: mean (standard deviation); Nominal data: *N* (percentage)

WSES = World Society of Emergency Surgery, AKI = acute kidney injury, LOS = length of stay, ICU = intensive care unit

Minor = WSES Grade I, Moderate = WSES Grade II or III, Severe = WSES Grade IV

*Student *t* test, ^†^Chi-square test

## Discussion

### Validation of the WSES classification using nationwide real-world data

In 2017, a novel classification of pelvic fracture that considered both the anatomy of the injury and physiological status was published by the WSES [8]. Different from previous classifications based only on mechanical fracture patterns, this classification also considered hemodynamic stability. With these guidelines, the evaluation of outcomes became more accurate, and critical decisions could be made more effectively. The results of the current nationwide study validated the reliability and feasibility of the 2017 WSES classification system (Table 1). Furthermore, two points (associated vascular injury and open fracture) that could potentially reinforce this guideline were also observed.

### The association of vascular injury and pelvic ring stability in the WSES classification

Per the WSES classification, minor and moderate injuries were distinguished using mechanical stability. It has been suggested that unstable pelvic fractures are associated with a higher probability of retroperitoneal hemorrhage because they tend to be associated with higher energy. It was reported that the incidence of major ligament disruption-related vascular injury ranged from 18 to 62.5% [13–15]. Therefore, unstable pelvic fracture is usually thought to be a more severe pelvic injury. However, vascular injury was also found in 7–10% of stable pelvic fractures, which have often been considered minor injuries and are normally treated conservatively [14, 15]. Some of these fractures even require angioembolization to achieve hemostasis [9, 14, 16]. Not only mechanical stability but also associated vascular injury should be evaluated in the management of pelvic fracture. Wu et al. reported that vascular injury severity is more closely correlated with the outcome than the type of anatomical fracture of the pelvis [9]. Our previous study, which was based on a single institution experience, also showed that the role of pelvic stability is not significant when evaluating associated hemorrhage in the management of patients with pelvic fracture [17].

In the univariate analysis of the current study, nonsurvivors had a significantly higher proportion of unstable pelvic fractures than survivors (Table 2). MLR analysis revealed that the effect of pelvic stability on the mortality of pelvic fracture was nonsignificant (Table 3). However, associated vascular injury served as an independent risk factor for mortality after adjusting for other covariates. Furthermore, even though patients were classified as having the same WSES class, patients with vascular injuries had significantly worse outcomes than patients without vascular injuries (Table 4). A subgroup analysis of pelvic fracture patients with vascular injuries was performed. In these patients, there was no significant difference in pelvic stability between survivors and nonsurvivors (Table 5). The above results showed that the association between pelvic stability and mortality could not be observed in the current study. In other words, it is not enough to evaluate the outcomes of pelvic fracture classified only in terms of mechanical stability. Associated vascular injury needs to be considered in the evaluation of pelvic fractures.

Per the NTDB dictionary, some patients with vascular injuries may not be extracted. Most patients with pelvic ring injuries (80–90%) with hemodynamic instability show venous bleeding, especially derived from the presacral venous plexus. Only approximately 10% of patients have bleeding from the arterial origin that might be recorded by the ICD-CM classification as vascular injuries (the most often identified sources of arterial bleeding are the internal iliac artery and its branches) [18, 19]. Therefore, although vascular injury plays an important role in the evaluation of pelvic injury, it only presents in the minority of cases. However, most venous bleeding can be managed conservatively, whereas arterial bleeding usually needs hemostasis procedures such as angioembolization or preperitoneal packing. In other words, it makes sense that patients with vascular injuries that were recorded in the NTDB had a poorer outcome.

### Open/closed pelvic fracture in the WSES classification

In contrast to closed fractures, open pelvic fractures can lead to concomitant external hemorrhage, internal hemorrhage, associated anorectal or urogenital injuries and contaminated wound-related infection [3, 20, 21]. Several complications, such as septicemia, AKI and multiple organ dysfunction, may occur because of an uncontrolled infection [22]. A review article by Grotz and coworkers concluded that urethral and vaginal injuries were the most common urogenital injuries in open pelvic fracture, present in 24–57% of patients [3]. Song et al. reported a study of twenty open pelvic fracture patients with rectal injuries, 50% of whom had pelvic infection [23]. Thus, the high mortality rate of open pelvic fracture, which is related to septicemia and multiple organ dysfunction, has been reported by several studies. Frane et al. observed an in-hospital mortality rate of 11.6% in a total of 19,834 open pelvic fracture patients [24]. Tseng et al. showed a 21.6% mortality rate in 37 open pelvic patients [21]. However, although it is an easily identified but life-threatening lesion in the ED, open pelvic fracture was not evaluated and discussed in the 2017 WSES classification.

In the current study, open pelvic fracture patients had significantly worse outcomes (higher mortality rate, higher infection rate, longer hospital LOS and longer ICU LOS) than closed pelvic fracture patients for all WSES classes (minor, moderate and severe) (Table 2). The proportion of patients with AKI, which is usually associated with severe infection and sepsis, was also significantly higher in the moderate and severe classes. Moreover, the role of the WSES classification appears to be different in patients with closed and open pelvic fractures (Fig. 1: *R*^2^ of closed fracture = 0.8848, *R*^2^ of open fracture = 0.9809). Therefore, it is better not to mix closed and open fractures in the same classification system. Open fractures should be evaluated independently.

### Limitations of the current study

The NTDB data are retrospective, incomplete and possibly inaccurate. Without detailed records regarding resuscitation and imaging findings, associated vascular injury and unstable pelvic ring injury could only be defined based on the coding system. In addition, the WSES classification uses multiple parameters to determine hemodynamic instability, including SBP < 90 mmHg, pulse > 120/min, evidence of skin vasoconstriction (cool, clammy, decreased capillary refill), altered level of consciousness and/or shortness of breath. However, there was no record of physical examination or blood transfusion amount in the NTDB 2015. Some parameters that were used to determine hemodynamic instability could not be analyzed. We understand that it is insufficient to define unstable hemodynamics using parameters in the NTDB. This is a potential weak point of nationwide big data. We tried our best to find patients who fulfilled definitions of unstable hemodynamics (SBP, pulse or use of vasopressor from the PCODE). However, there may be some patients with missed coding or classification. We believe that a trend toward accurate outcome prediction by the WSES classification could be observed using a real-world database. Furthermore, to evaluate outcomes, it is better to design a study with isolated pelvic fracture patients only. These patients were analyzed for the validation of WSES classification (Table 1). However, pelvic ring injury patients with vascular injuries or open pelvic fracture usually have other extrapelvis-associated injuries [1–3]. For other analyses, the patient number would be small, and statistically significant results would not be achieved. Therefore, we excluded severe head injuries that usually affect outcomes and included the ISS in the MLR model to eliminate the effect of other nonpelvic injuries. Finally, the research datasets were not updated. Several techniques have been developed in the management of pelvic injuries, such as preperitoneal packing and resuscitative endovascular balloon occlusion of the aorta [25, 26]. However, the treatment was not discussed in the current study. The clinical evaluation has not changed much. We believe that the data from 2015 are applicable to current trauma patients.

## Conclusion

The WSES guideline provides an accurate and reproducible classification of pelvic fractures according to this nationwide study. It is recommended that open/closed fractures and associated vascular injuries be evaluated as supplements of the WSES classification.

## Data Availability

The data and material were from a public databank (National Trauma Data Bank).

## Abbreviations

WSES: World Society of Emergency Surgery
AKI: Acute kidney injury
ED: Emergency department
NTDB: National Trauma Data Bank
ICD: International Classification of Diseases
AIS: Abbreviated injury scale
SBP: Systolic blood pressure
RR: Respiratory rate
GCS: Glasgow Coma Scale
ISS: Injury severity score
LOS: Length of stay
ICU: Intensive care unit
MLR: Multivariate logistic regression
